# The Perkin-Elmer approach to computer-aided chemistry: an overview of the technological trends in laboratory computing

**DOI:** 10.1155/S1463924684000146

**Published:** 1984

**Authors:** Stephen A. Reber, John P. Coates

**Affiliations:** The Perkin-Elmer Corporation M.S. 56 and M.S. 905 Norwalk Connecticut 06856 USA


Journal of Automatic Chemistry, Volume 6, Number 2 (April–June 1984), pages 67–73

### The Perkin-Elmer approach to computer-aided chemistry: an overview of the technological trends in laboratory computing

**Stephen A. Reber and John P. Coates**

*The Perkin-Elmer Corporation, M.S. 56 and M.S. 905, Norwalk, Connecticut 06856, USA*

Perkin-Elmer defines the term 'computer-aided chemistry' as the application of a computer, of any size, to the solution of an analytical chemistry problem. More specifically, the computer is a *tool* which is applied to tasks of either a tedious, repetitive, or very complex nature so that the analytical chemist is freed to apply his reasoning power. This paper was prepared for a session on 'Solving the laboratory management problem' at the 1983 Pittsburgh Conference and the authors were asked to contemplate the future laboratory applications for this extremely important tool. The time allotted was 30 min, so the article focuses on only the most significant applications among those that Perkin-Elmer is working on.

### Three levels of computer hardware

Computer technology can be defined as spanning three levels of hardware: the dedicated microprocessor; the stand-alone general-purpose microcomputer; and the minicomputer. A microcomputer fits on a desk top, whereas a minicomputer typically does not. Actually, the categories are not that simple. The 'dedicated microprocessor' class includes stand-alone units that are dedicated to working with an instrument; that is, collecting raw analytical data and reducing it. At the minicomputer end of the spectrum, there are 16-bit minicomputers and 32-bit superminis. The definition of 'minicomputer' used here refers to 32-bit superminis, primarily because the increased power of 16-bit micros (some with 32-bit internal architecture) and the rapidly declining price of 32-bit superminis will probably cause the 16-bit mini to disappear over the next few years.

One could also define a fourth level—mainframes. Many laboratories use mainframes for time-sharing, complex modelling, payroll and accounting functions, archiving etc. Since this paper is limited to *dedicated* laboratory computing, mainframes are not included. Now that supermini prices are lower than those of many laboratory instruments, minis are rapidly supplanting time-shared mainframes in laboratories.

This paper's focus is on levels 2 and 3 because that is where the technological change is fastest. However, it is relevant to touch on the subject of the microprocessor.

The decade of the 70s was the microprocessor era. Its advent was so rapid that today, as we all know, almost every instrument offered by every company contains one. The microprocessor has had the greatest impact of any single technological innovation on the proliferation of analytical chemistry applications. Perhaps the most obvious reason is that its ability to distill raw data has allowed certain techniques—for instance, mass spectrometry—to become more widely applicable. More important, however, has been the microprocessor's ability to *automate* analyses, thereby reducing costs. A comparison of costs for a typical gas-chromatography analysis over the 10-year period between 1972 and 1982 indicates that not only is the instrument much cheaper, excluding inflation, but the variable cost per analysis differs by a factor of *six*. This tremendous cost reduction triggered a series of events leading to a great increase in the importance of analytical chemistry:

Lower costs (and regulatory requirements)
↓
More applications and more demand for analyses
↓
More demand for automation
↓
Even lower costs etc.

Many laboratory managers with whom laboratory information management has been discussed have indicated that their sample load increased by a factor of 10 or more during the 70s, yet the total headcount did not even double.

Microprocessors are becoming more powerful and so they will continue to lower costs. However, the era of the microprocessor is coming to a close, and the era of the micro and mini is just beginning.

### Definitions

Before going any further, several terms need to be defined. First, there are two classes of laboratory data:

(1) *Management information*: sample load, backlog of samples, turnaround time and cost per analysis for example.
(2) *Analytical or scientific data*, for example data from an instrument.

It is also necessary to define the concepts of *distributed and centralized processing.*

'Centralized processing' means that a minicomputer collects raw data from instruments, through an analogue-to-digital converter, processes the data, and produces reports (see figure 1). An example is Perkin-Elmer's Chromatography Laboratory Automation System (C/LAS), which can collect data from more than 70 chromatographs simultaneously, reduce the data, and store results within a data-base. Essentially, it is a huge, multichannel integrator.

'Distributed processing' is a buzzword which can have several definitions. There are two significant meanings that apply to laboratory computing. First, distributed processing has

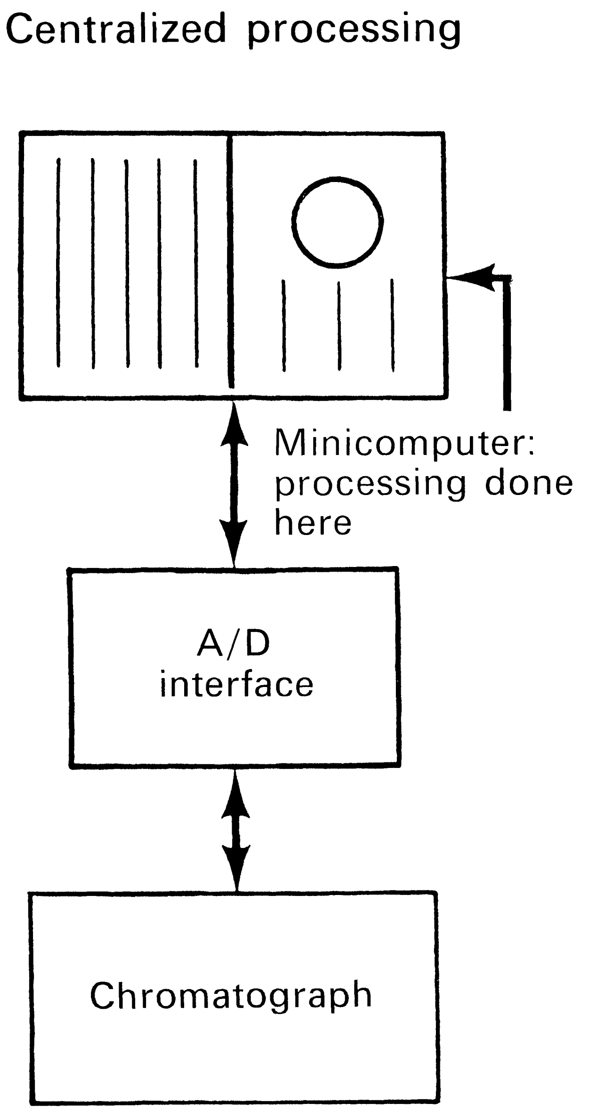

*Figure 1. Example of centralized processing.*

*Table 1. Relative advantages in favour of centralized and distributed processing.*

| *Centralized system advantages* | *Distributed system advantages* |
|---|---|
| Often cheaper, since each A/D interface is less expensive than a microcomputer | Microcomputer does data collection and reduction, off-loading minicomputer processor |
| 'Cookbook' parameters can be stored on minicomputer disk for automated data collection/reduction | Worker has a microcomputer at his disposal; hence, more flexibility |
| | The software for analytical calculations is more extensive on microcomputers For instance Perkin-Elmer offer more than 40 packages, and the user library programs number over 60. |
| | Processor redundancy: if the minicomputer goes down, the laboratory can continue operating |

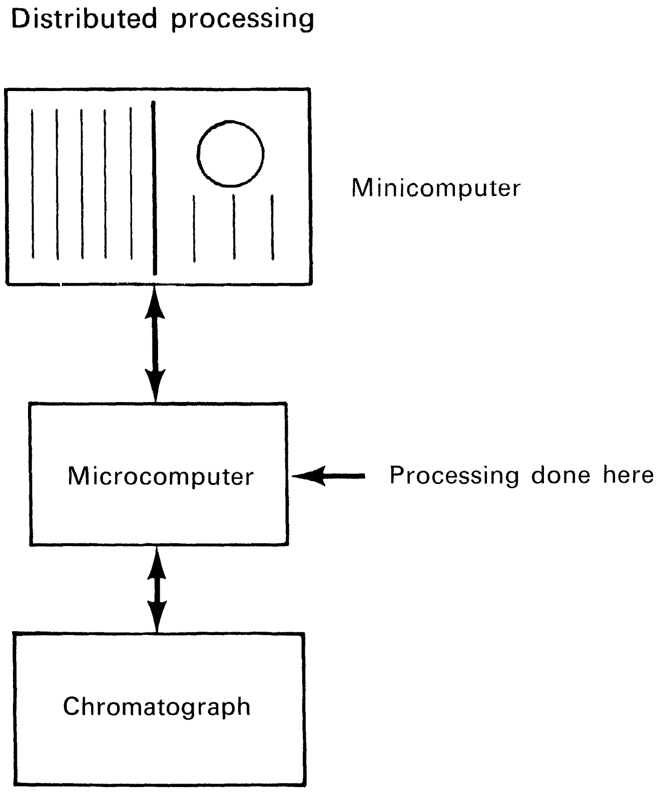

*Figure 2. Example of distributed processing.*

a meaning at the minicomputer level, in that a superminicomputer is now powerful enough to do many jobs that were previously possible only on a mainframe, yet low enough in price to be dedicated to a laboratory's use. A supermini system, complete with software, now starts at about $80 000. When the supermini is dedicated to a laboratory's use, several problems with mainframes disappear, including time-sharing with other users, complex operating systems, heavy software overhead, and working entirely through the company's MIS (Management Information Systems) staff to get a program written. The second definition of distributed processing pertains to performing analytical data collection and reduction on microcomputers, as opposed to minicomputers (see figure 2). The trend toward this type of distributed processing, which has significant ramifications for laboratory computing, is discussed later in this paper. Table 1 presents some of the arguments in favour of both centralized and distributed analytical data processing.

For the remainder of this paper, the term 'distributed processing' will refer to the second definition above.

### Types of laboratories and computing applications

In this section, four general types of laboratories are defined and the differences among their computing needs are described. In reality, laboratories do not fall neatly into these categories; however, they are useful for the purpose of this discussion. The four classes are as follows:

(1) *Quality control* (QC): mostly routine analyses; require turnkey analytical software packages; high sample throughput; high demand for data storage; increasing demand for management information; tend to buy minicomputers for multichannel data collection/reduction.

(2) *Research & development* (R&D): mostly non-routine analyses; require flexible computing systems; low sample throughput; tend to buy micros for dedicated (one-on-one) use with instruments.

(3) *Analytical services*: a combination of QC and R&D.
(4) *Contract labs*: offer analytical services to other companies; requirements similar to QC labs.

According to surveys at Perkin-Elmer, analytical laboratories are distributed among these four categories approximately as follows:

| | |
|---|---|
| QC | 25% |
| R&D | 30% |
| Analytical services | 35% |
| Contract | 10% |

The QC and R&D labs are discussed further later in this paper because they represent the two extremes of the computing spectrum.

### The changing roles of micros and minis

As stated above, QC labs have historically tended toward using minicomputers for centralized processing (i.e. data collection/reduction), especially for chromatography. There have been several reasons for this, primarily capital cost savings, but also because of the ability to store raw data easily and to store methods in one place for use by all the laboratory's analysts. Obviously, this is much easier on one hard disk than on dozens of floppies or cassette-tapes.

Several technological trends are now causing these arguments in favour of minis to disappear. First, consider the argument that the minicomputer reduces costs. One can now purchase a perfectly adequate single-channel chromatography integrator for \$3000–\$5000. If your laboratory has 20 chromatographs, you can buy 20 integrators for \$60 000–\$100 000, which is considerably less than the price of a complete minicomputer system for 20 chromatography channels.

Next, consider the argument in favour of raw data storage. Microcomputers are now shipped with 10 megabyte disks, and 20 MB will be routine within the next year. Two years ago the maximum disk storage on the most popular minicomputer for chromatography was 20 MB.

The earlier discussion of QC labs pointed out the high sample throughput and the resulting importance of data storage, methods storage, and the increasing demand for *management* information (presented earlier). These requirements can be satisfied by a properly implemented Laboratory Information Management System, or LIMS, which utilizes data-base management technology. This requirement is distinctly different from the huge multichannel integrator that has served QC labs in the past. The multichannel integrator has a 'real-time' component; that is, the processor must be available to service each analogue-to-digital converter as it becomes ready to pass data to the minicomputer, otherwise data will be lost. Therefore, the data-collection task must be a high-priority, or 'foreground' task. Conversely, the LIMS tasks—for example, accessing the data-base at a terminal to check on a sample's status or to print a worklist for a department—must be low-priority, or 'background', tasks. If a minicomputer is set up for both types of tasks, there must be additional software 'overhead'; one program services the data collection and schedules other programs for post-run data collection and reduction. Another set of programs satisfies the requests from terminals. There is a 'technological envelope' which describes the minicomputer's ability to collect data from numerous instruments, yet provide an adequate response time at any terminal. Even on a superminicomputer, an analytical laboratory can easily go beyond this envelope.

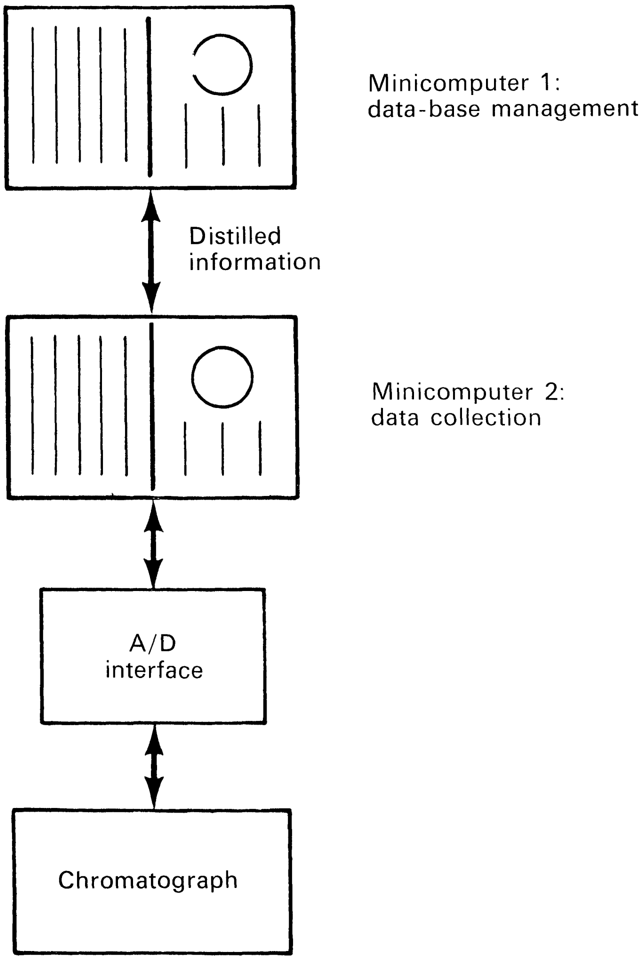

*Figure 3. Separating the real-time tasks and terminal requests by using two minicomputers.*

It is far preferable to *separate* the tasks of data collection and terminal requests by running them on separate processors. One obvious way to do this would be to collect data on one minicomputer, passing on the distilled information to a second minicomputer, which is providing management information (see figure 3).

A second means for separating the tasks is to do data collection/reduction on dedicated micros, which pass on the reduced data and, if desired, raw or processed data for long-term storage (see figure 2). In this configuration, it would be desirable to allow down-line loading of programs from the supermini to the micros. That is, a program could be compiled on the supermini, and the object code down-loaded and run on the micro, as required.

The second scheme has several important advantages over the first:

(1) A microcomputer at each instrument which allows greater flexibility for the operator, as needed.
(2) Processor redundancy. If the minicomputer fails, data collection can continue until the local disk fills (if the local disk is removable, data collection can proceed indefinitely). If a micro fails it can be replaced by another, since micros are relatively cheap.
(3) Lower cost. At this point in time, the initial capital outlay is lower, in most cases, to buy dedicated micros than to buy minicomputers for multichannel data-collection and reduction. Over the next few years, the gap will probably widen.

Since the arguments overwhelmingly favour distributed processing, we feel that the configuration shown in figure 2 will appear in most medium-to-large sized laboratories over the next five to 10 years. Smaller labs may find their needs satisfied by a dedicated supermini doing both data collection and data-base management.

The R&D lab, which lies at the other end of the computing spectrum, will also favour distributed processing. R&D personnel usually desire the flexibility of dedicated, programmable micros, because it is easier to adapt the computer to each type of sample—and the range of samples may be very wide. In the past, however, a minicomputer was often needed for power and for archiving.

The new high-end micros, especially those that are 68000-based, can now satisfy most of the 'power' requirements. Also, their operating systems are much easier to deal with than the OS of the mini.

R&D labs will continue to use minicomputers for cataloging and archiving samples (i.e. an electronic lab notebook), and for large-scale modelling and correlation studies. The ability to compile a program on a mini and down-line load it is obviously also important.

One of the keys to distributing data-processing—for both QC and R&D labs—will be data-communications technology, especially Local Area Networking (LAN), which is discussed later.

### Microcomputer trends

The increasing applicability of micros due to their rapidly increasing power and storage capabilities and diminishing cost has already been discussed. However, there is a lot more to micros than hardware. Perkin-Elmer feel very strongly that the major key to the proliferation of micros is *applications software.* This statement appears so frequently in journals, newspapers—even *TIME Magazine*—that it seems silly to repeat it. Yet, there are still many laboratory people who buy a computer based upon the hardware alone or upon the name tag. In the latter case, the hope is that the large company selling it has such a large user library that there will be a program *somewhere* that fits the application perfectly. This is seldom true, because user-library programs are notoriously inflexible, poorly documented and improperly debugged.

Within the past year, there has been a considerable move toward 'standardization' among the microcomputer vendors—especially on hardware. New micros—almost invariably—are based upon a Motorola 68000 or an Intel 808X. Though proceeding more slowly, there is also a move toward standardizing on operating systems: a UNIX OS or similar OS on the 68000 and CP/M-86 or MSDOS on the Intel chips. Due to the emerging standards on microcomputer hardware and systems software, hardware is fast becoming a commodity.

Application software, however, is far from a commodity. Over the past years, Perkin-Elmer has mounted a major effort to create applications software for analytical chemistry in all nine of the major product lines. The trends we see boil down to the following areas:

(1) *Ergonomics*—that is, software that is easy to use.
(2) *Graphics.*
(3) *Data management.*
(4) *'Ancillary' software*, for example word processing, spread sheet, and business graphics software.

Let's consider the first two topics together, because the subject of graphics is really a subset of the more general term 'ergonomics'. 'Ergonomics' refers to making microcomputers easy to use, both for information input and for display. A large fraction of the laboratory population still suffers from 'keyboard fear', and so one key to the proliferation of micros will lie in overcoming that fear. There are many approaches to accomplishing this, two of which are described here. The first is the use of 'hard' function keys. These keys can be hard-coded within the computer program to perform a specific function. The operator need not remember code names in order to perform a function—rather, he presses the appropriate key and the function is automatically called up. Hard function keys have been used for several years.

A newer example is the use of touch-sensitive, 'soft' keys. Associated with these keys is a row of eight blocks which are written on the screen itself. The computer program controls the function of each of the eight keys, and writes in the eight blocks on the screen a brief description of the function that will result from pressing the key. Once a key is pressed, another series of eight functions might be assigned by the program to the soft keys. Therefore, the program operates in much the same way as a menu-driven system. The difference is that the operator interacts directly with the screen instead of the keyboard, which helps overcome 'keyboard fear'.

A second example of ergonomic design is the appropriate use of graphics, an especially important subject in the laboratory. Recent advances in CRT technology and the rapid decline in the price of random-access memory are now making the use of colour practical. The use of colour will probably be the most important new introduction to graphics technology over the next several years because of the new dimension that it adds to the visual impact of a screen. The Perkin-Elmer Professional Computer 7500 allows the simultaneous display of up to 16 colours from a palette of 27. The aesthetic effect alone contributes to the system's ergonomic value. However, there are many other benefits, where numerous spectra or chromatograms must be compared, where a base-line assignment must be visualized, and so forth.

### *Data management*

As stated earlier, microcomputers now contain large disks—10 MB or more. This means that a large amount of data can be stored. It is obviously important to properly organize that data on the disk for recall, and so data management software will be incorporated with software for analytical chemistry in the future.

### *Ancillary software*

Over the past year, we have discovered a remarkable increase in the demand for business-computer-type software, especially word processing. Analytical laboratories do a great deal of report generation, and so word processing heads the list.

The meaning of 'word processing' to a scientist differs from the meaning to a secretary in that the package must be extremely easy to use and, generally, less sophisticated. Other important packages for general use include data management (for general record-keeping), spread sheet packages (for example VISICALC) for manipulating rows and columns of numbers, statistics and business graphics (pie charts, histograms etc.). Most laboratory microcomputer vendors will probably offer such packages over the next few years.

### Minicomputers

We will now leave the microcomputers and move on to level 3—the superminicomputer.

In defining the roles of the micro and the supermini, we arrived earlier at several tasks to which the supermini will be applied in analytical laboratories:

(1) Laboratory information management.
(2) Raw data storage or archiving.
(3) Program and methods storage for down-line loading.
(4) Program development and compilation.
(5) Complex modelling.
(6) Number-crunching that requires the supermini's power.

As discussed before, data collection may be included in this list in smaller laboratories.

The least straightforward item in the list above—and also the item that will have by far the greatest impact on analytical labs—is laboratory information management.

### *Laboratory Information Management Systems (LIMS)*

A LIMS is a data-base management system designed specifically for a laboratory and running on a supermini. An example is Perkin-Elmer's LIMS/2000, which has now been on the market for two years. Its goal is to harness the flow of information in a laboratory so that the laboratory's primary goals can be achieved: high analysis quality, fast sample turnaround, and the productive use of resources. Each sample is logged into the system as it enters the laboratory, and, as each analysis is completed, the results go into the data-base.

The benefits that accrue from a LIMS are enormous, as indicated by the list in table 2. Obviously, these benefits far transcend those gained from the traditional use of a minicomputer for collecting and reducing analytical data.

*Table 2. Some of the benefits that result from the implementation of a LIMS.*

(1) Automatic sample numbering
(2) Standardized sample log-in menus
(3) Verification of information entered
(4) Automatic test assignment for routine samples
(5) Automatic generation of a receipt for the submitter
(6) Automatic production of labels
(7) Generation of a report on sample distribution
(8) Generation of worklists with samples in the correct order
(9) Generation of work orders
(10) Automated data collection and reduction for chromatography and other techniques
(11) Automatic comparison of results with an acceptable range
(12) Prompting filing of standard tests and methods
(14) Easy inspection of previously collected data
(15) Easy time charge entry
(16) Backlog reports for scheduling purposes
(17) Exception reports
(18) Easy inspection of results for supervisor's approval
(19) Sample and test status checks
(20) Sample location tracking
(21) Rapid response to submitter inquiries
(22) Reports on performance, by department:
Turnaround time
Productivity
Quality
(23) Non-routine searches for information
(24) Automatic report generation
(25) Word processing
(26) Long-term filing of data, or archiving
(27) Easy graphical display and manipulation
(28) On-line programming capability
(29) Inspection of work profiles by customer, test, or sample type
(30) Workload projections
(31) Cost allocations
(32) Automatic billing
(33) Reduction in paperwork
(34) Reduction in errors
(35) Increased ability to answer inquiries from higher levels of management

Now that powerful 32-bit hardware is available for less than the cost of some analytical instruments, the key to the future of laboratory information management lies in the *applications software*. Most 32-bit minis already offer excellent operating systems and data-base management packages. However, these 'tools' must be combined with higher-level software that makes it easy to create the benefits listed in table 2. Figure 4 illustrates the software structure of Perkin-Elmer's LIMS/2000, which contains a very flexible *user interface* which buffers the applications software from the systems software.

### Data communications

Communications technology is currently undergoing tremendous change. Due to the proliferation of computers, the ability to send data from one point to another is now as important to most companies as sending audio signals.

Over the next several years, standards will emerge in three areas that affect analytical laboratories:

(1) Benchtop standards—i.e. controlling instruments and autosamplers and controlling data collection where cables are no more than 50 ft long.
(2) Local Area Networks (LAN)—communications among members of one department or in one building.
(3) Global networks—communications among large computers, typically over great distances (including trans-oceanic communication).

Because benchtop systems have existed for many years, instrument companies have already moved towards standards in that area. The most widely offered communications accessory is RS-232C (serial asynchronous). RS-232C allows rates effectively up to 9600 bit/s, which is adequate for many applications. Over the past few years there has been a discernible trend toward IEEE-488, which is capable of much higher rates. IEEE-488 is expected to become more common in benchtop systems.

Local Area Network (LAN) technology, the most familiar example being Ethernet, is now approaching standardization. LANs are critical to the proliferation of distributed processing due to several very attractive features:

(*a*) Only one cable is necessary into which micros and terminals can be plugged, thus affording flexibility.
(*b*) Very high data rates (10 million bits/s) are possible.
(*c*) Communications between any two nodes along the network are possible, without routing the message through the minicomputer, thus relieving its processor of a substantial burden. Two examples of the use of this feature include electronic mail (i.e. one user sends electronic messages to another) and shared resources (that is, sharing peripherals such as expensive printers or plotters). At this moment, the cost per connection is fairly high (approximately $1000); however, when a standard develops, the cost will plummet.

Global networks will offer many advantages, including the following:

(i) A user in one location can access a data-base in another location.
(ii) Messages or data files can be sent over large distances.
(iii) A user in one location can run a program that exists only in another location.

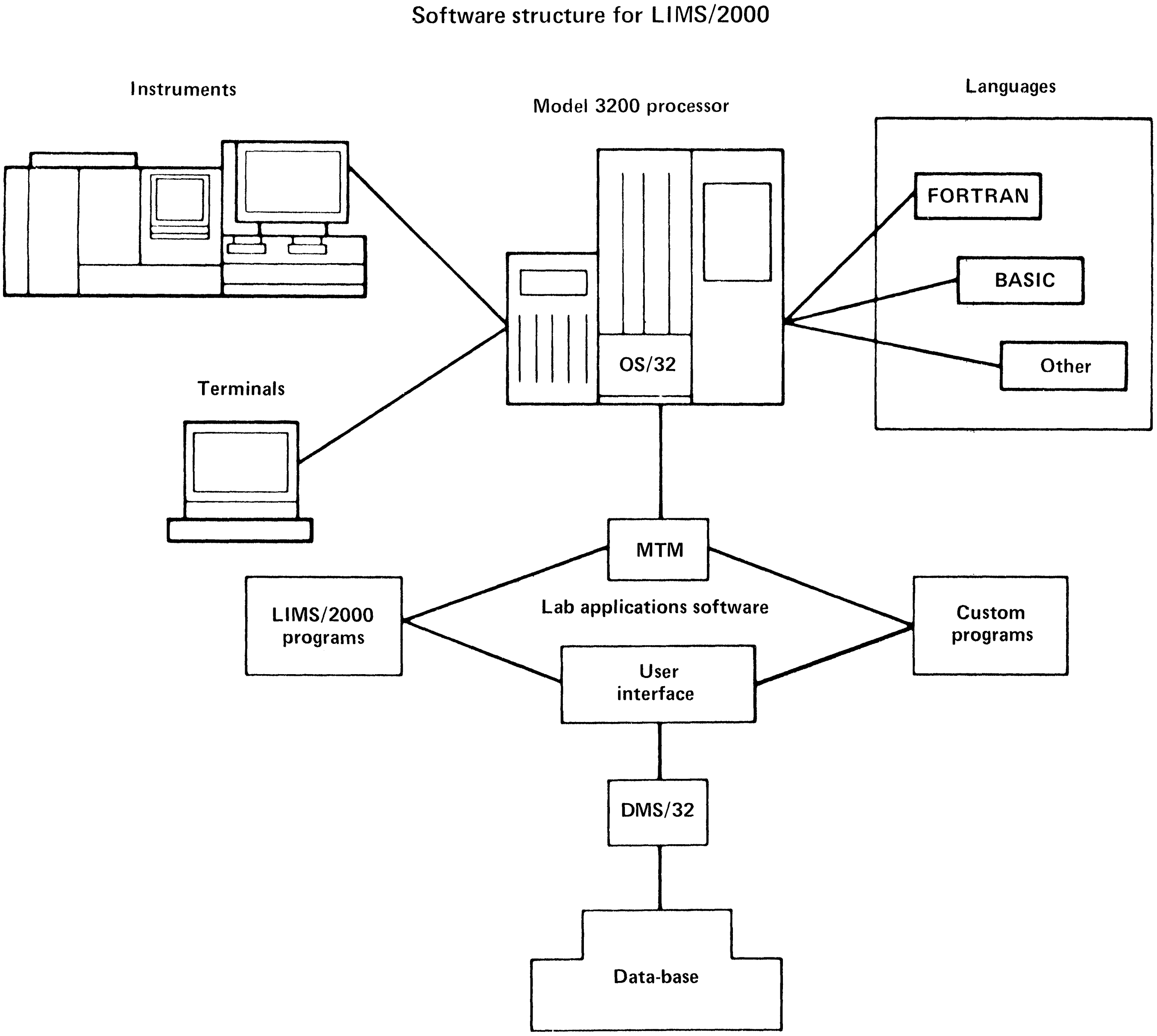

*Figure 4. The software structure of LIMS/2000.*

*Table 3. Examples of LRP reports.*

| *Operational* | *Strategic* |
|---|---|
| Sorted worklist | Invoice |
| Backlog profile | Backlog history |
| Work order | Throughput history |
| Exception report | Turnaround time measurement |
| Sample location tracking | Time-charge measurement |
| Automatic or manual time-charging | Time-charge summary |
| Automatic promise date assignment | Usage totals |

### Laboratory Resource Planning (LRP)

In the area of applications software, one key to the future lies in what Perkin-Elmer terms 'Laboratory Resource Planning', or LRP. LRP can be defined as a series of reports that enhance the ability of laboratory people to make tactical and strategic decisions that affect the *management* of the lab. That is, LRP reports help ensure that samples are analysed in the correct order, sample turnaround time is measured in a meaningful way, sudden changes in the backlog or in work-load trends are flagged immediately, and so forth. One simple example of an LRP report can indicate when an analytical backlog is present and provide a report broken down by department, test, sample age and priority level. Table 3 lists some typical LRP reports. LRP really represents a whole new technology. It is analogous to Material Resource Planning (MRP), which has been developed for factories over the past two decades.

### Conclusion

This paper has covered a very broad range of technologies. The changing roles of minicomputers and microcomputers have been considered, with minis doing mostly data-base management and micros handling real-time tasks; that is, a distributed processing architecture. The emerging importance of applications software for specific analytical techniques has also been covered, as well as its ergonomic design and the use of colour graphics. Also discussed were the subjects of Laboratory Information Management Systems (LIMS) and Laboratory

Resource Planning (LRP). Finally, the key to the distributed architecture—data communications—was considered, with the proliferation of Local Area Networks (LANs) being a key issue.

One of the interesting implications of the discussion in this paper is that the trends noted above are analogous to those of the 'office-of-the-future', where professional, secretarial and clerical personnel work at and communicate through personal workstations. The laboratory seems to be evolving toward the same type of system architecture where clerks, analysts, chemists, supervisors and managers work at personal *scientific workstations* and each instrument has a dedicated workstation as well, while the minicomputer serves as an electronic filing-cabinet.

The potential changes noted above certainly will not occur overnight. However, it does appear that the next five to 10 years will bring substantial productivity increases to analytical laboratories and so the excitement of the 1970s will continue.

### COLLOQUIUM SPECTROSCOPIUM INTERNATIONALE XXIV

*To be held from 15 to 21 September 1985 in Garmisch-Partenkirchen, FR Germany*

CSI XXIV will touch upon all areas of analytical chemistry; an international group of spectroscopists is expected to attend. Programme topics (conference languages are to be English, French and German) were recently announced:

*Basic theory and methods—*

- Atomic emission spectroscopy
- Atomic absorption spectrometry
- Atomic fluorescence spectrometry
- X-ray emission and fluorescence spectrometry
- Methods of surface analysis and depth profiling
- Infra-red and Raman spectroscopy
- Molecular spectroscopy (UV and Vis)
- Radiation detectors, data recording and handling, automation
- Laser spectroscopy
- Mass spectroscopy (organic and inorganic)
- Standard reference materials etc.

*Analytical applications for specific problems—*

- Analysis of metals
- Analysis of different industrial products
- Geochemical analysis
- Biological, clinical and pharmaceutical analysis
- Analysis in agriculture and nutrition chemistry
- Environmental analysis.

*The second circular will soon be available from CSI XXIV, Organisationsbüro, Institut für Spektrochemie und angewandte Spektroskopie, Postfach 778, D 4600 Dortmund 1, FR Germany.*

### ANALYTICON 84

*4–6 September 1984, The Barbican Centre, London*

Analyticon, once again, will have the support of The Royal Society of Chemistry and The Chromatographic Society (previously The Chromatography Discussion Group) and will be staged alongside LABORATORY 84.

The conference is intended for scientists and technologists from all branches of industrial and medical science and related academic disciplines where analytical techniques play a major role and where technological advances in the field of instrumentation and computers can be considered.

The subjects to be covered at Analyticon will fall into two parallel 'streams'. The programme will be arranged, however, so that plenary lectures can be attended by all conference delegates attending each day and topics of common application and interest will, as far as possible, be staggered. Time will also be allowed to attend possible sessions within the conference programme.

Each of the six main themes listed below covers several subject areas:

- Computers in the laboratory
- Chromatography
- Molecular characterization and surface analysis
- Elemental analysis
- Clinical analysis and biosciences
- Electroanalytical methods.

All delegates to the conference will have free access to the exhibition halls plus an invitation to attend social events.

The conference will be opened by the new President of The Royal Society of Chemistry: Professor R. O. C. Norman.

*For full programme and registration details contact Mr G. C. Young, The Scientific Instrument Manufacturers' Association, Leicester House, 8 Leicester Street, London WC2H 7BN. Tel.: 01 437 0678.*

### PRODUCT DESIGN ASSURANCE IN ENGINEERING

*11–13 June 1985, London*

The Society of Environmental Engineers' 1985 conference at Wembley will cover Specifications, Techniques and Case studies.

In conjunction with the conference there will be a major exhibition of environmental equipment.

*Details from Helen Gibbons, Society of Environmental Engineers, Owles Hall, Buntingford, Hertfordshire, UK.*

